# Transplant Trial Watch

**DOI:** 10.3389/ti.2023.11742

**Published:** 2023-07-27

**Authors:** John Matthew O’Callaghan

**Affiliations:** ^1^ University Hospitals Coventry and Warwickshire, Coventry, United Kingdom; ^2^ Centre for Evidence in Transplantation, University of Oxford, Oxford, United Kingdom

**Keywords:** kidney transplant, randomised controlled trial, mTOR inhibitor, cardiovascular outcomes, CMV infection

To keep the transplantation community informed about recently published level 1 evidence in organ transplantation ESOT and the Centre for Evidence in Transplantation have developed the Transplant Trial Watch. The Transplant Trial Watch is a monthly overview of 10 new randomised controlled trials (RCTs) and systematic reviews. This page of Transplant International offers commentaries on methodological issues and clinical implications on two articles of particular interest from the CET Transplant Trial Watch monthly selection. For all high quality evidence in solid organ transplantation, visit the Transplant Library: www.transplantlibrary.com.

**RANDOMISED CONTROLLED TRIAL 1**

Cardiovascular Outcomes in *De Novo* Kidney Transplant Recipients Receiving Everolimus and Reduced Calcineurin Inhibitor or Standard Triple Therapy: 24-Month Post Hoc Analysis From TRANSFORM Study.

*by Sommerer, C., et al. Transplantation 2023 [record in progress]*.

## Aims

This *post hoc* analysis of the TRANSFORM study aimed to compare the effect of everolimus (EVR) and reduced calcineurin inhibitor (rCNI) versus standard triple therapy on cardiovascular disease (CVD) outcomes in *de novo* kidney transplant patients.

## Interventions

Participants in the TRANSFORM trial were randomised to either the EVR + rCNI group or the mycophenolic acid (MPA) + standard-exposure CNI (sCNI) group.

## Participants

2026 *de novo* kidney transplant recipients.

## Outcomes

The main outcomes of interest were the incidence of major adverse cardiac event (MACE), cardiac deaths, time-to-event analysis of MACE, CVD risk factors and levels of metabolic parameters.

## Follow-Up

24 months.

## CET Conclusion

Everolimus is known to worsen post-transplant dyslipidaemia, but it is not clear that this results in poorer cardiovascular outcomes. This *post hoc* analysis of the TRANSFORM study, which compared outcomes in kidney transplant patients on standard immunosuppressive therapy vs. a everolimus/reduced tacrolimus regimen, compared the rate of major adverse cardiac events (MACE) between the two groups over a 2 year follow up period. Over 2,000 patients were included in the analysis. Although lipid levels were increased in the everolimus group as expected, the rate of MACEs were not significantly different between the two groups. The authors speculated whether this could be due to cardio-protective effects due to everolimus, which had previously been demonstrated in preclinical studies, and which offset the lipid dysregulation effects. With the potential to reduce posttransplant viral infections, these findings provide further evidence to support everolimus based regimens as a viable alternative to current immunotherapy standard of care.

## Trial Registration

Clinicaltrials.gov—NCT01950819.

## Funding Source

Industry funded.

**RANDOMISED CONTROLLED TRIAL 2**

Conversion to mTOR Inhibitor to Reduce the Incidence of Cytomegalovirus Recurrence in Kidney Transplant Recipients Receiving Preemptive Treatment: A Prospective, Randomized Trial.

*by Viana, L. A., et al. Transplantation 2023 [record in progress]*.

## Aims

The investigators aim to assess if switching from mycophenolate or azathioprine to sirolimus following first cytomegalovirus (CMV) infection post-transplant reduces recurrence rate of CMV infections.

## Interventions

Once randomised the intervention group were abruptly converted from antimetabolite (mycophenolate sodium 720 mg twice daily or azathioprine 2 mg/kg once daily) to sirolimus (5–8 ng/mL), tacrolimus was continued, but the maintained concentrations were lowered to 3–5 ng/mL. The control group continued tacrolimus (5–10 ng/mL) and their antiproliferative agent.

## Participants

72 adult kidney transplant recipients who had a treated CMV infection within the first 6 months after transplant.

## Outcomes

The primary endpoint was the incidence of recurrent CMV infection within 12 months following randomisation. The secondary endpoints were: Incidence of *de novo* DSA, kidney function, proteinuria, acute rejection, graft loss and death.

## Follow-Up

Participants were followed-up for 12 months.

## CET Conclusion

This small prospective unblinded randomised control trial demonstrates that conversion to sirolimus form antimetabolite following initial CMV infection has a significant effect to reduce recurrent infection. Within the sirolimus cohort no episodes of CMV infection or disease occurred within the study period. Whereas the control group had a recurrence rate of 43%. All the patients in this study are high risk for CMV with all recipients being CMV positive pre-operative, but received no pre-emptive pharmacological treatment, simply weekly blood monitoring. Upon CMV infection/disease intravenous ganciclovir was commenced and once treated the antimetabolite switch to sirolimus and the study period commenced. Within the study group they saw no significant difference in biopsy confirmed acute rejection, *de novo* DSA, proteinuria, graft survival or death. The generalisability of these findings is limited due to the strict inclusion criteria, within their study period they randomised 72 patients out of a total of 1,309 with a first-treated CMV infection/disease, all of whom were low-immunological risk. The patient cohort and small sample size limits safety and efficacy conclusion as well as conclusion in broader recipient populations, such as D+/R−. While there is insufficient evidence to change current practice it sits along side other trials, some of which multi-centre and larger supporting mTORi in improving CMV related outcomes, although often with higher discontinuation rates.

## Jadad Score

3.

## Data Analysis

Strict intention-to-treat analysis.

## Allocation Concealment

Yes.

## Trial Registration

ClinicalTrials.gov—NCT02671318.

## Funding Source

Industry funded.

## Clinical Impact Summary

Pre-emptive treatment of CMV after renal transplantation is associated with a considerable risk of later CMV infection recurrence, and further changes to immune suppression and acute rejection. Sirolimus and other MTORi are associated with lower risk of CMV infection.

The study is a small but interesting one and of good quality. Patients could be included after the first episode of CMV infection or disease, and were then randomised to convert to sirolimus or stay on mycophenolate/azathioprine. Tacrolimus was continued in both groups.

There was no recurrence of CMV in the group randomised to sirolimus, which is in stark contrast to the 43% recurrence in the control arm. Importantly there was no significant difference in the incidence of treated biopsy proven acute rejection within 12 months after randomisation.

This study provides convincing evidence for a potential method to tailoring immune suppression and reduce the risk of further complications for CMV. However, the population was highly selected to be at low risk of rejection, and that needs to be kept in mind.

